# TRACE (Trunk Aesthetic Clinical Evaluation), a routine clinical tool to evaluate aesthetics in scoliosis patients: development from the Aesthetic Index (AI) and repeatability

**DOI:** 10.1186/1748-7161-4-3

**Published:** 2009-01-20

**Authors:** Fabio Zaina, Stefano Negrini, Salvatore Atanasio

**Affiliations:** 1 ISICO (Italian Scientific Spine Institute), Via Roberto Bellarmino 13/1, 20141 Milan, Italy

## Abstract

**Background:**

Aesthetic appearance is of primary importance in the treatment of adolescent idiopathic scoliosis (AIS), but to date tools for routine clinical practice have not become available. The aim of the present study is to develop such a tool and to verify its repeatability.

**Methods:**

Instrumentation: At first we developed the Aesthetic Index (AI), based on a three-point scale for asymmetry of the shoulders, scapulae and waist that we tested for 5 years. From this experience we developed another tool we called TRACE, the acronym of Trunk Aesthetic Clinical Evaluation; TRACE is a 12-point scale based on four sub-scales, shoulders (0–3), scapulae (0–2), hemi-thorax (0–2) and waist (0–4).

Population: Posterior-anterior (PA) photographs of one hundred-sixty AIS patients

Procedures: Each photograph was scored in two independent tests by four observers using AI, and subsequently TRACE.

Data analysis: Kappa statistical analysis and 95% level of agreement were used; we also identified the minimum significant change (95% confidence level).

**Results:**

We found the intra- and inter-raters repeatability of AI to be fair. Three points out of seven was the minimum significant change between two different evaluations. For TRACE, intra-rater repeatability was fair and inter-raters poor; but the minimum significant change was three (intra-rater), or four (inter-raters) out of twelve points.

**Conclusion:**

Widening the scale from 7 (AI) to 12 points (TRACE) increased the clinical sensitivity to changes of the aesthetic scale, even if TRACE has only a fair repeatability. TRACE is a no-cost tool for routine clinical practice in AIS patients. Due to the absence of other comparable validated tools, once the inherent measurement error is known and understood, its routine clinical use by physicians is advised.

## Background

Aesthetic appearance is a primary consideration in the treatment of scoliosis. This has been clearly stated in a consensus by SOSORT experts, in which aesthetic improvement has become the main goal of scoliosis treatment.[1] Orthopaedic surgeons share this view of the relevance of aesthetics; in a recent study concerning the importance of physical deformity of patients with adolescent idiopathic scoliosis, "the severity of deformity" consistently ranked as the most important clinical consideration when proposing surgical treatment to patients.[2] Some attempts to measure and monitor aesthetics have been made. Some questionnaires, such as SRS 22,[3] include domains concerning aesthetics, while some questionnaires have been designed and validated specifically to measure the perception of spinal deformity by the patient (or the parent). This is the case of the Walter Reed Visual Assessment Scale [4] and the more recently developed "Spinal Appearance Questionnaire" [5]. These instruments have the advantages of considering the patient's subjective judgement of his own aesthetics, but this does not correspond to the objective situation as can be judged by an external observer. This means that these are more psychological than aesthetic evaluation tools.

Various high-tech instruments for trunk surface evaluation are available, such as ISIS, Formetric, Quantec, AUSCAN and others [6-12], but none has reached any kind of consensus or is used extensively in routine clinical practice. This is particularly attributable to the high cost of such instruments, which limits availability to hospitals and clinics while hindering the integration of these instruments into standardized procedures. Attempts have been made to establish the inter-rater reliability of aesthetics clinical evaluation in AIS patients, but the results have not been satisfactory [13]. Thus, despite the judgment among physicians that aesthetics is of great importance in AIS patients, there are no clinical practice tools by which to assess its changes during treatment.

The objective of the present study was to develop an routine clinical tool and verify its intra- and inter-rater repeatability in the assessment of aesthetics in AIS patients.

## Methods

For more than twenty years our group has evaluated the aesthetics of the posterior trunk, ranking the asymmetry of the shoulders, scapulae and waist (0 absent, 1 slight, 2 important) even if without giving to this evaluation a great importance. Facing the needs reported in the introduction, in the last five years we set out to build on our experience to develop a new clinical tool called the Aesthetic Index (AI), which corresponds to the sum of these three subscale scores.

### Population

Posterior-anterior (PA) photographs of one hundred-sixty AIS patients

### Procedures

Each photograph has been scored twice independently by four observers. In this way we had four pairs of intra-rater and four pairs of inter-rater evaluations. Each single observer performed the evaluation twice, and there was an interval of one week between observations.

### Data analysis

We used Kappa statistics (0–0.2 poor, 0.2–0.4 fair, 0.4–0.6 moderate, 0.6–0.8 good, 0.8–1.0 very good), an index of the observer disagreement which compares the agreement found against that which might be expected by chance. Kappa can be thought of as the chance-corrected proportional agreement, and possible values range from +1 (perfect agreement) via 0 (no agreement above that expected by chance) to -1 (complete disagreement). Despite published controversies [14-16], Kappa statistics are still widely used. In particular, we are aware that Kappa may be low even though there are high levels of agreement and even though individual ratings are accurate. Whether a given kappa value implies a good or a bad rating system or diagnostic method depends on what model one assumes about the decision making of raters [17]. Accordingly we also used the Percent of agreement, the percentage of the answers that were equal in the two repeated measures. Moreover, for clinical purposes, we present the 95% level of agreement, or the number of points of difference needed to reach an agreement of 95%. To give an example: for the rater who obtained the worst results the Percent of agreement for TRACE was 28.8% (Table 1), but if the repeated measurements one point above (or below) are considered, a 99.4% of agreement can be reached. This corresponds to a 95% of agreement of 2 points out of 12. This result has an high clinical significance, because it means that, in everyday practice, considering two evaluations made by the same rater, a real change occurs only if the variation is over the 95% level of agreement, that in the example given corresponds to 3 points out of 12.

**Table 1:** Results of statistical analysis of TRACE and its individual items

	Kappa statistics range		Percent of agreement		95% level of agreement (range)		Minimum Significant Change	
	
	Intra-raters	Inter-raters	Intra-raters	Inter-raters	Intra-raters	Inter-raters	Intra-raters	Intra-raters
TRACE	0.16–0.24	0.09–0.14	28.8–36.3	18.8–36.1	2/12 (99.4–96.9%)	3/12 (95.0–100%)	3/12	4/12

Shoulders	0.29–0.43	0.16–0.25	51.3–70.6	48.8–70.6	1/4 (96.9–100%)	1/4 (92.5–100%)	2/4	2/4

Scapulae	0.43–0.58	0.41–0.50	76.9–79.4	70.6–80.0	1/3 (99.4–100%)	1/3 (100–100%)	2/3	2/3

Hemi-thorax	0.22–0.41	0.12–0.20	58.8–63.1	50.6–63.1	1/3 (98.1–99.4%)	1/3 (95.6–99.4%)	2/3	2/3

Waist	0.40–0.48	0.07–0.11	55.0–68.0	24.4–68.1	1/5 (95.6–99.4%)	2/5 (98.7–100%)	2/5	3/5

## Second Study: TRACE

Given the results from the first part of our study, we developed a new scale called TRACE, that is the acronym of Trunk Aesthetic Clinical Evaluation), for the purpose of improving the AI widening (and deepening) the scale.

### Instrumentation

**Figure 1:**
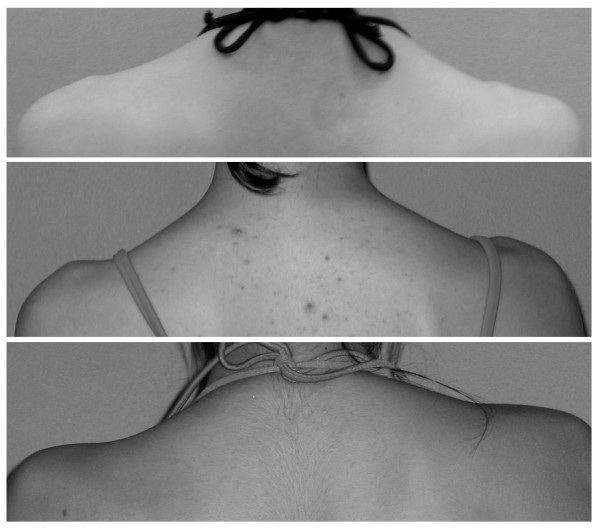
**Shoulder asymmetry, as evaluated in TRACE, ranges from 0 to 3**. For the shoulders it is easy to detect some intermediate values, so we defined asymmetry (from the top) slight (1), moderate (2) and important (3).

**Figure 2:**
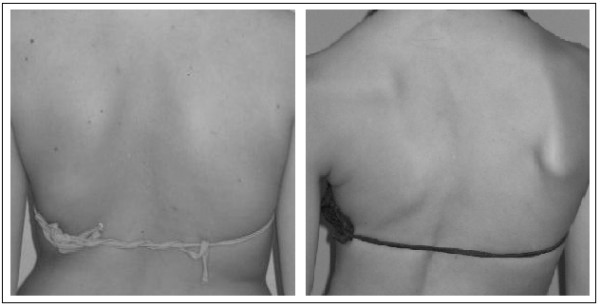
**Hemi-thorax asymmetry as evaluated in TRACE**: This item was created as a complement of the scapulae, since we noted that occasionally there is an evident prominence of the last ribs on the back even when there is no real asymmetry in the scapulae. From the left: slight (1) and important (2) asymmetry.

**Figure 3:**
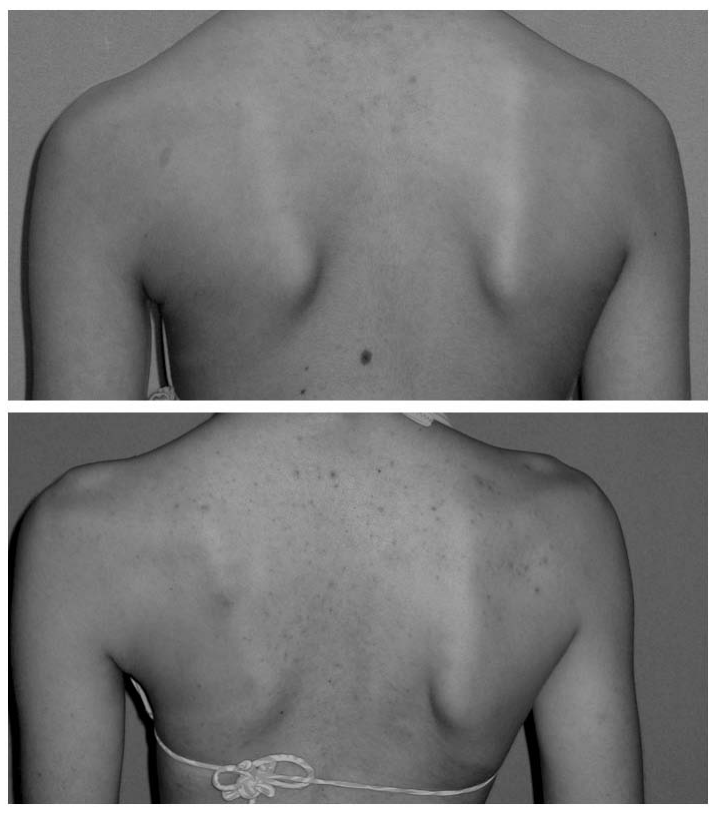
**Scapulae asymmetry as evaluated in TRACE**: (from the left) slight (1) and important (2).

**Figure 4:**
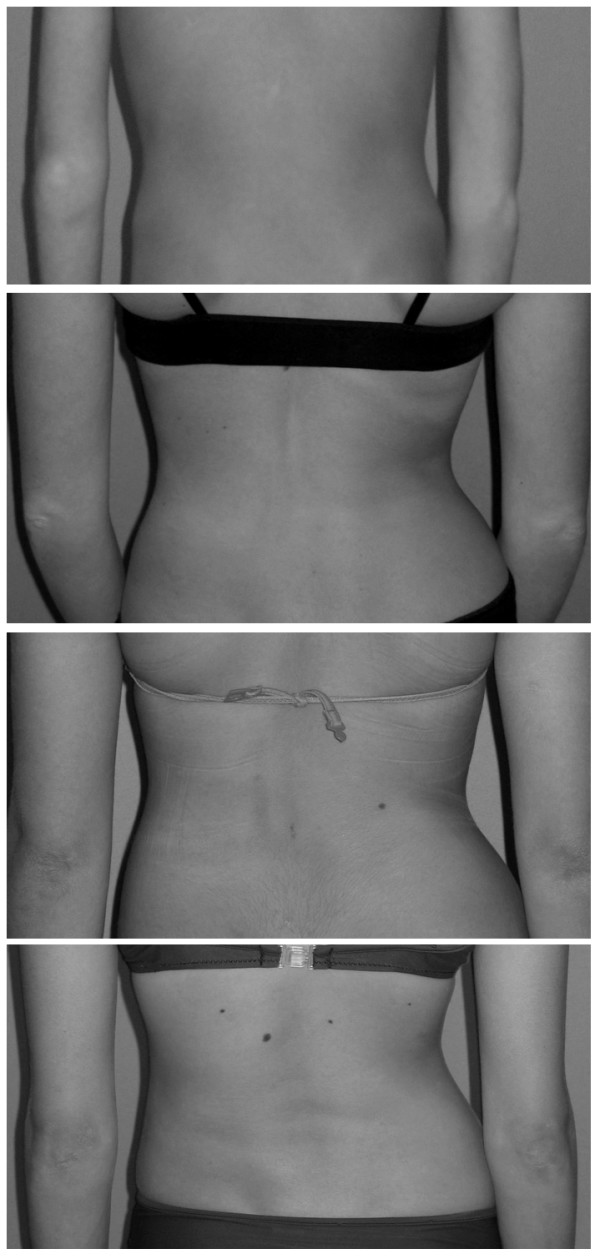
**Waist asymmetry as evaluated in TRACE**: it was quite easy to define a total asymmetry (a score of 4) when one flank was straight or when there was a lateral decompensation of the trunk. It was easy as well to define a very slight (a score of 1) and an important but not complete (a score of 3) asymmetry; between these points we defined a mild asymmetry (a score of 2). In the figure, from the top: slight (1), mild (2), moderate (3) and important (4) asymmetry.

TRACE is based on four sub-scales: shoulders, scapulae and waist (which were already present in the AI), and the hemi-thorax (Fig 1, 2, 3, 4). However, the scores for each sub-scale were changed with respect to AI: shoulders now ranged from 0–3, waist from 0–4, scapulae from 0–2 and hemi-thorax from 0–2. From these sub-scales we calculated TRACE, using the sum of the sub-scale scores to reach a 12-point scale. These changes were based on our experience in using the AI. We realized that for the shoulders it was easy to define more intermediate values, so we defined asymmetry as slight (1), moderate (2) or important (3). For the waist it was easy to define a total asymmetry (a score of 4) when one flank was straight or when there was a lateral decompensation of the trunk. It was easy as well to define a very slight (a score of 1) and an important but not complete (a score of 3) asymmetry. Between these points we defined a mild asymmetry (a score of 2). The hemi-thorax item was created as a complement of the scapulae, since we noted that occasionally there is an evident prominence of the last ribs of the back even when there is no real asymmetry in the scapulae.

### Procedures

The testing procedure of TRACE was similar to that of AI: The same 160 PA photographs of the trunks of AIS patients were evaluated by the same observers through the use of the same procedure.

### Data analysis

The same statistical analysis used for AI was performed. We also correlated the AI scores with TRACE by comparing the values of the shoulders and waist, as well as the overall value of TRACE. The scapulae were not included in the analysis because the scores did not change.

## Results

Regarding the AI, we found the repeatability of both intra- and inter-raters to be fair (range of Kappa value 0.28–0.41 and 0.17–0.28 respectively). The waist was the more reproducible sub-score, rating good and fair, respectively, for intra- and inter-raters (Table 2). At the 95% level of agreement, we found that three points out of seven was the minimum change to be considered significant between two different evaluations both for the same and different raters.

**Table 2:** Results of statistical analysis of the Aesthetic Index and its individual items.

	Kappa statistics range		Percent of agreement		95% level of agreement (range)		Minimum Significant Change	
	
	Intra-raters	Inter-raters	Intra-raters	Inter-raters	Intra-raters	Inter-raters	Intra-raters	Inter-raters
Aesthetic Index	0.28–0.41	0.17–0.28	46.2–55.6	37.6–41.5	2/7 (98.7–99.4%)	2/7 (92.5–99.4%)	3/7	3/7

Shoulders	0.42–0.53	0.30–0.35	64.4–73.7	58.3–61.2	1/3 (99.4–100%)	1/3 (96.9–100%)	2/3	2/3

Scapulae	0.50–0.58	0.20–0.43	69.4–76.9	53.5–61.9	1/3 (98.1–100%)	1/3 (95.6–100%)	2/3	2/3

Waist	0.48–0.70	0.28–0.33	75.6–82.5	62.8–66.1	1/3 (100–100%)	1/3 (98.7–100%)	2/3	2/3

Widening the scale with TRACE, we found intra-rater repeatability to be fair, while inter-raters were poor (Kappa value: 0.16–0.24 and 0.09–0.14 respectively; Table 1). All sub-scores graded as moderate for intra-rater, while the inter-rater was lower (moderate for the scapulae, poor for the waist and fair for the other subscores). At the 95% level of agreement, we found that three points out of twelve was the minimum change to be considered significant between two different evaluations for the same rater, while four out of twelve was the minimum for different raters.

The correlations between the AI and TRACE are shown in the tables 3, 4 and 5.

**Table 3:** Correlation between Trace and AI values for the shoulder

AI	TRACE					Corresponding TRACE value
		
	0	1	2	3	TOT	
0	16%	70%	14%	1%	100%	0.99

1	4%	52%	38%	7%	100%	1.48

2	0%	7%	33%	60%	100%	2.53

**Table 4:** Correlation between Trace and AI values for waist.

AI	TRACE						Corresponding TRACE value
		
	0	1	2	3	4	TOT	
0	16%	73%	11%	1%	0%	100%	0.96

1	1%	35%	37%	20%	6%	100%	1.94

2	1%	1%	15%	44%	39%	100%	3.20

**Table 5:** Correlation between Trace and AI total values

AI	TRACE													Corresponding TRACE value
		
	1	2	3	4	5	6	7	8	9	10	11	12	TOT	
0	0%	17%	38%	42%	4%	0%	0%	0%	0%	0%	0%	0%	100%	2.33

1	0%	2%	15%	30%	28%	12%	9%	2%	1%	0%	0%	0%	100%	3.73

2	0%	0%	4%	19%	27%	26%	14%	6%	1%	1%	0%	0%	100%	4.56

3	0%	0%	2%	6%	20%	26%	24%	12%	7%	3%	1%	0%	100%	5.44

4	0%	0%	0%	1%	6%	15%	29%	25%	14%	4%	4%	1%	100%	6.56

5	0%	0%	0%	0%	0%	2%	14%	9%	22%	22%	30%	2%	100%	8.44

6	0%	0%	0%	0%	0%	0%	14%	0%	0%	7%	64%	14%	100%	9.50

## Discussion

With this paper we aimed to develop a routine clinical tool for aesthetic evaluation of scoliosis patients, evolving from our yearly experience in grading some parameters (shoulders, scapulae, waist).

The goal was to verify the intra- and inter-rater repeatability, but most of all the sensitivity to change in a clinical setting. A secondary aim was to build on our experience to develop a new tool to be studied. Our first evaluation showed an overall fair repeatability for AI. Buchanan et al. [13] found a similar reliability for the intra- and inter-observer cosmetic deformity rating among a group of orthopaedic surgeons. However, the present study revealed a low sensitivity of AI to changes; indeed, a three-point change out of seven is the minimum change that could be considered significant. This limits the application of the AI to the detection of major changes. Therefore, we broadened all applicable parameters, determined through our experience to be easily detectable, and developed TRACE. While maintaining the same fair intra-rater repeatability, a higher sensitivity to changes is the main feature of TRACE. In fact, the 95% level of agreement remained similar (2 points both for AI and TRACE for intra-rater) but the scale was now of 12 instead of 7 points (almost double that of AI): A score of three points out of twelve represents a significant change during treatment when the observer is the same. This makes TRACE much more useful than AI, since it makes it possible to objectively monitor the aesthetic effects of treatment. Both AI and TRACE have been used as research tools, and we documented that TRACE is sensitive enough to detect changes induced by a brace treatment [18-20]. Moreover, this is a "no-cost" tool that can be used easily and quickly during each clinical assessment. It requires neither expensive instruments nor prolonged evaluation sessions. Usually it is sufficient to mark the sub-scale values and calculate the total TRACE score, such that in routine clinical practice photographic comparison is not needed (as we usually do since 5 years with our ISICO database software) [19,21-23].

Trunk deformity significantly influences AIS patients' perception of function and self-image [24]. Therefore, both rehabilitation experts and surgeons emphasise this aspect in the decision-making process in AIS [1,2,25]. To date, the main outcome measures concerning the aesthetic effects of treatment are related to prominence changes [26] and the improvement of vertebral rotation [27] after brace treatment, to reduced Cobb angle after surgery [28,29], or the improvement in self-perception of the deformity as assessed by questionnaire.[4] Some attempts to quantify aesthetic deformity with a clinical assessment have been performed: Theologis proposed a "Cosmetic Spinal Score (CSS)," according to which ten non-medical judges evaluated colour pictures of AIS patients [8]. The limit of this evaluation is that it gives a score pertaining to a general impression of the patient's back but is not based on precisely defined sub-scores. Nevertheless, CSS was shown to be related principally to rib hump and trunk side shift. Moreover, we have no data concerning its reliability when performed by expert physicians.

The principle that scoliosis is not simply a curvature indicated through x-ray imaging and that there is a need for appropriate outcome measures to supplement Cobb angle has been widely recognized [1,25]. TRACE provides a semi-quantitative scale for clinical assessment of deformity in AIS, based on specifically defined sub-scales. Knowing the limits and the repeatability of this scale will give clinicians more reliability in the routine clinical assessment of deformity, and will provide other sensitive outcome measures. TRACE is consistent with this need, and it can readily be used in the clinical evaluation of AIS patients and for research.

One limitation of this study was the use of pictures instead of an immediate evaluation of patients. Nevertheless, evaluation through pictures has been the standard applied in previous studies,[2,4,5] and we can presume the repeatability to be even greater during routine clinical practice due to the opportunity for a three-dimensional evaluation of the patient. In fact, photographs are static while a three-dimensional clinical assessment presumably can be more consistently recorded by the physician, and in future studies can provide a tool to compare TRACE with the POTSI index. Another limit could be the low Kappa Statistics values obtained, even if comparable to those obtained by others evaluating aesthetics previously[8]; but is less important for clinical routine use than the minimum significant change.

This study documents the evolution of TRACE from AI. TRACE is sufficiently repeatable and sensible for routine clinical practice, and therefore comprises is a no-cost tool designed for the conservative clinical setting.

## Competing interests

The authors declare that they have no competing interests.

## Authors' contributions

All authors made substantial contributions to conception, design and acquisition of data; they have been involved in drafting and revising the manuscript; they have given final approval of the version to be published.
